# Preparation and in vitro antibacterial evaluation of gatifloxacin mucoadhesive gellan system

**Published:** 2010

**Authors:** K. Kesavan, G. Nath, JK. Pandit

**Affiliations:** 1 Department of Pharmaceutics, Institute of Technology; 2 Department of Microbiology, Institute of Medical Science, Banaras Hindu University, Varanasi, Uttar Pradesh, India

**Keywords:** Mucoadhesive, Mucin, Rheological, Gellan gum, Sodium carboxymethyl cellulose, Sodium alginate

## Abstract

**Background and the purpose of the study:**

The poor bioavailability and therapeutic response exhibited by the conventional ophthalmic solutions due to precorneal elimination of the drug may be overcome by the use of mucoadhesive *in situ* gel forming systems that are instilled as drops into the eye and undergo a sol-gel transition in the cul-de-sac and have good mucoadhesion with ocular mucus layers. The objective of this study was to formulate ophthalmic mucoadhesive system of gatifloxacin (GTN) and to evaluate its in vitro antibacterial potential against, *Staphylococcus aureus* and *Escherichia coli*.

**Methods:**

: Mucoadhesive systems were prepared using gellan combined with sodium carboxymethylcellulose (NaCMC) or sodium alginate to enhance the gel bioadhesion properties. The prepared formulations were evaluated for their gelation, and rheological behaviors, mucoadhesion force, in vitro drug release, and antibacterial activity.

**Results:**

All formulations in non-physiological or physiological conditions showed pseudoplastic behaviors. Increase in the concentration of mucoadhesive agent enhanced the mucoadhesive force significantly. In vitro release of gatifloxacin from the mucoadhesive system in simulated tear fluid (STF, pH of 7.4) was influenced significantly by the properties and concentration of gellan, sodium carboxymethyl cellulose and sodium alginate. Significant reduction in the total bacterial count was observed between drug solution (control) and mucoadhesive batches against both tested organisms.

**Major conclusion:**

The developed mucoadhesive system is a viable alternative to conventional eye drops of GTN due to its ability to enhance bioavailability through its longer precorneal residence time and ability to sustain the release of the drug.

## INTRODUCTION

Topical delivery of eye drops is the most common practice of drug treatment in ocular diseases. The sites of action of most of the ophthalmic drugs are located in the inner eye. Although, external eye structures are readily accessible, the biological barriers, mainly the corneal epithelium, limit ocular drug absorption (1, 2).

Ocular therapy would be significantly improved if the precorneal residence time of drugs could be increased. Several new preparations have been developed for ophthalmic use, not only to prolong the contact time of the vehicle on the ocular surface, but also to slow down drug elimination (3, 4). Successful results have been obtained with inserts (4) and collagen shields (5). However, these preparations also have some disadvantages, such as poor compliance, especially by elderly people, and loss of the device in some patients without noticing it. From the view point of acceptability many patients prefer, a liquid dosage form.

Mucus is a viscous and heterogeneous biological product that coats many epithelial surfaces (6). Mucus-secreting cells are widely spread in different locations in the body, including the nasal, ocular and buccal area and the gastrointestinal, reproductive and respiratory tracts.

Mucoadhesive drug delivery systems utilize bioadhesive property of certain water-soluble polymers that become adhesive to mucous membranes on hydration (7) and hence can be used for targeting a drug to a particular region of the body for an extended period of time (8).

The idea of mucoadhesives was derived from the need to localize drugs at a certain site in the body. The extent of drug absorption is limited by the residence time of the drug at the absorption site. Particularly, in ocular drug delivery, less than 2 min is available for drug absorption after instillation of a drug solution into the eye and since the drug is removed rapidly by solution drainage hence the ability to extend contact time of an ocular drug delivery system in front of the eye would undoubtedly improve drug bioavailability.

Gatifloxacin, a fourth generation fluoroquinolone is used to treat bacterial conjunctivitis (9). It is the drug of choice in the treatment of bacterial infections and available as eye drops and eye ointments in the market. Newer formulation components are being explored to develop a comprehensive and controlled release strategy. Utilization of the principle of sustained release as embodied by mucoadheisve systems therefore offers an attractive alternative approach to the difficulties of prolonging pre-corneal drug residence time.

Sodium CMC and sodium alginate were selected as mucoadhesive polymers due to their better mucoadhesive capacity compared to other mucoadhesive polymers like poly (acrylic acid) (PAA) and polycarbophils. PAA adhesion is very sensitive to the presence of ions and the shielding of the carboxyl group by cations present in the tear fluid diminish the interaction of PAA with the functional group on mucin (10). Polycarbophil shows the maximum adhesion strength at pH of 3, decreasing gradually by increasing pH, towards 5, above which it does not show any mucoadhesivity.

A combination of gellan with sodium alginate or sodium CMC would be very promising for ocular administration as a mucoadhesive system. The result could be a prolonging of the contact time. In fact, mucoadhesive *in situ* gelling ocular inserts of gatifloxacin (11), have been already studied. For better patient acceptability, a liquid dosage form that can sustain drug release and remain in contact with the cornea for extended periods of time is an ideal option. Our objective was to develop a mucoadhesive system of gatifloxacin using gellan alone and in combination with sodium alginate or sodium carboxymethylcellulose which would undergo gelation when instilled into the cul-de-sac of the eye and provide prolonged retention on the external ocular surface.

## MATERIAL AND METHODS

Gatifloxacin (GTN) and Gellan gum were the kind gifts by Burgeon Pharmaceutical Pvt. Ltd., (Kancheepuram, India) and ISP (Hong Kong Ltd., Hyderabad, India), respectively. Sodium alginate and sodium carboxymethyl cellulose were kind gifts of Dr.Reddy's laboratory (Hyderabad, India). Benzalkonium chloride was purchased from RFCL Ltd. (New Delhi, India). Mucin type II and Cellulose membrane were purchased from Sigma- Aldrich chemicals pvt. Ltd., New Delhi. Mannitol was purchased from Sisco Research Lab. (Mumbai, India). Sodium Chloride, Sodium acetate, Calcium chloride (2H_2_O) and Glacial acetic acid were purchased from Merck (Mumbai, India). Sodium bicarbonate was purchased from SD fine chemical Ltd. (Mumbai, India). All other reagents were of analytical grade.

### 

#### Preparation of formulations

Gellan alone and in combination with sodium alginate and sodium carboxymethylcellulose (Table 1) was dissolved in hot acetate buffer of pH 5.0 (70°C, prepared in fresh water for injection under laminar flow) by continuous stirring at 40°C. Then 300mg of gatifloxacin was added to the polymeric solution and stirred until dissolved. Mannitol and benzalkonium chloride which were used as isotonicity agent and a preservative, respectively, were added later. The formulations were filled in sterile 100 ml amber coloured bottles, capped with rubber closures and sealed with aluminum caps. The formulations, in their final pack were terminally sterilized by autoclaving at 121°C and 15 p.s.i. for 20 min. The sterilized formulations were stored in refrigerator (4–8°C) until further use.

**Table 1:** Components and evaluation of mucoadhesive system of gatifloxacin.

S. No	Batch code	Drug (% w/v)	Gellan (% w/v)	Sodium alginate (% w/v)	NaCMC (% w/v)	Drug Content (%±S. D.)^a^	Gelling Capacity in STF^a^	Mucoadhesion Force (dyne/cm^2^)^a^
1	Control	0.3	-	-	-	99.58± 1.05	-	-
2	GG_1_	0.3	0.015	-	-	99.42±0.86	+	6.18±1.61
3	GG_2_	0.3	0.03	-	-	98.54±1.04	++	13.36±1.39
4	GG_3_	0.3	0.03	-	0.1	99.48±1.2	+++	30.56±2.28
5	GG_4_	0.3	0.03	-	0.25	101.1±0.95	+++	44.26±3.75
6	GG_5_	0.3	0.03	-	0.5	98.87±0.52	+++	66.13±2.46
7	GG_6_	0.3	0.045	-	-	99.24±0.41	+++	19.54±1.73
8	GG_7_	0.3	0.06	-	-	99.18±0.64	+++	22.04±2.84
9	GM_1_	0.3	0.03	0.4	-	98.85±0.95	+++	29.39±1.51
10	GM_2_	0.3	0.03	0.6	-	99.45±0.76	+++	40.58±3.34
11	GM_3_	0.3	0.03	0.8	-	100.06±0.82	+++	60.45±3.43

All formulation containing 5% Mannitol and 0.02% benzalkonium chloride were used as isotonic agent and preservative, respectively.

+ -Gels after few minutes

++ -Gels immediately but remains for a few hrs (less stiffer)

+++ -Immediate gelation and remains for extended periods and formed gels are stiffer.

a Values reported as mean±SD (n=3)

#### Evaluation of the formulations

#### Drug content uniformity

The bottle (n=3) containing the preparations was shaken for 2–3 min and by a micropipette 100 µl of the preparation was transferred aseptically to sterile 25 ml volumetric flasks and the final volume was made up with Simulated Tear Fluid (Sodium chloride, 0.67 g, Sodium bicarbonate, 0.20 g, Calcium chloride (2H_2_ O), 0.008 g, Distilled water, q.s. 100 ml). The concentration of GTN was determined at 287 nm (Shimadzu, UV-1601, Japan).

#### Gelation studies

The gelation studies were carried out in gelation cells, fabricated locally using Teflon. The cells were cylindrical reservoirs capable of holding 3 ml of the gelation solution (STF). Within the cells located at the bottom was a 250 µl transparent plastic cup to hold the gel sample in place after its formation. Carefully 100 µl of the preparation was placed into the cavity of the cup using a micropipette and 2 ml of the gelation solution was added slowly. Gelation was deducted by visual examination.

#### Rheological studies

Viscosity evaluations of the prepared GTN formulations were carried out using Brookfiled DV- 111+ Rheometer with spindle LV-3. The viscosity of the formulations, either in non-physiological (pH of 5.0) or Physiological conditions (pH of 7.2) was determined using a 50 ml aliquot of the sample. Viscosity of samples were measured at different angular velocities from 10 to 100 rpm with an equal wait for 3 min in each rpm. The angular velocity was reversed (100 to 10 rpm). The average of the two readings was used to calculate the viscosity.

#### Mucoadhesive evaluation

A simple viscometric method was used to quantify mucin-polymer mucoadhesive strength (12). Viscosities of 15% (w/v) porcine gastric mucin dispersions in STF were measured with a Brookfield viscometer in the absence (η _m_) or presence (η _t_) of different formulations at 37°C and a shear rate of 100 rpm Viscometric measurements were performed after exactly 3 min of applying the shear force for homogeneous distribution throughout the sample. Viscosity components of mucoadhesion (η_b_) were calculated from the equation, η_t_=η_m_+η_p_+η_b_, where η_p_ is the viscosity of corresponding pure polymer solution. The force of mucoadhesion (F) was calculated from the equation, F=η_p_.σ, where σ is the rate of shear/sec.

#### In vitro release studies

The in vitro release of GTN from the formulation was studied through cellulose membrane using modified apparatus (13). The freshly prepared STF was used as dissolution medium. Cellulose membrane (molecular weight cut off 12,000 D, Sigma-Aldrich Chemicals, India), previously soaked overnight in the dissolution medium was tied to one end of a specifically designed glass cylinder (open at both ends). One ml of the formulation (equivalent to 3 mg of GTN) was mixed with 3 ml of STF and placed into this assembly. The cylinder was attached to a stand and suspended in 50 ml of dissolution medium maintained at 37±1°C and the membrane just touching the receptor medium surface. The dissolution medium (STF) was stirred with a star headed magnetic bead at 50 rpm. Aliquots of 5 ml volume were withdrawn at regular time intervals and replaced with an equal volume of the pre-warmed medium. The samples were analyzed for GTN content at 287 nm using UV spectrophotometer (Shimadzu-1601, Japan). The in vitro release was studied for the control (without polymer) in order to compare the release profile with the prepared.mucoadhesive system of gatifloxacin.

#### Mechanism of drug Release

In order to elucidate kinetics of the drug release, data were analyzed using: zero-order equation, Q=Q_0_+Kt;first-order equation, Q=Q_0_ e^-kt^ (14, 15) and Higuchi's square root model, Q=K√t (16). Where, Q is the amount of drug released in time “t”. Q_0_ is the initial dose, K is. the release constant of the respective equations, and t is the release time. In order to find out the drug release mechanism from the mucoadhesive systems, the data were fitted to Korsmeyer-Peppas’ power equation (17, 18): M_t_ /M_∞_=Kt^n^ where, M_t_ /M_∞_ is the fraction of drug released in time “t,” K is a constant incorporating structural and geometrical characteristics of the drug/ polymer system, and n is the release exponent, which is indicative of the drug-release mechanism. When n is equal to 0.5, the drug is released from the polymer with Fickian diffusion mechanism. If 0.5<n<1, it indicates anomalous or non-Fickian release, whereas if n=1, it indicates zero-order release.

#### Antimicrobial efficacy studies

#### Determination of minimum inhibitory concentration (MIC)

The MIC is defined as the lowest concentration of the antibiotic that inhibits all visible growth and was determined by serial dilution method (19). *Staphylococcus aureus* (ATCC 25923) and *Escherichia coli* (ATCC 25922) were used as gram-positive and gram-negative micro organisms, respectively*.*

The stock solution of gatifloxacin in both standard and test were prepared in the concentration of 3 mg/ ml in acetate buffer (pH of 5.0). Fifteen sterile test tubes were arranged in a rack and numbered from 1 to 15. From the stock solution serial dilution was made using Lubria (LB) broth to give concentrations of 76.5 µg/ml to 0.02 µg/ml placed in 1^st^ tube to 13^th^ tube. One tube was considered as the positive control and another tube as the negative control. Following subcultures from frozen stock, 10 µl broth of the standard *S.aureus* and *E.coli* containing 1x10^6^ CFU/ ml strains were inoculated in all test tubes except in negative control and incubated at 37°C for 24 hrs to observe growth. The tubes were observed for inhibition of growth and MIC was determined.

#### In vitro antibacterial activity

The in vitro antimicrobial sensitivity of the formulation was examined in vitro by a standard procedure (19). One milliliter of the sample (GG_5_) was mixed with 100 ml of STF solution for gelation. Then one milliliter of the above solution was added to a set of test tubes containing cultures of standard *E.coli* and *S.aureus* (1x10^6^ CFU/ml) in LB broth. In another set of experiment drug solution equivalent to 30 µg was used. The test tubes were incubated at 37±0.2°C, with continuous shaking. At 1,2,3,4,5,6,7 and 8 hrs time intervals, bacterial growth was determined by reading optical density at 600 nm (OD 600). Positive and negative controls were maintained throughout the study for comparison. The entire test was performed in triplicate and the values corresponded to average±S.D.

#### Interaction studies

Interaction studies were conducted by FT-IR and DSC to investigate any interaction between drug and excipients.

#### IR Study

The drug or polymer or freeze dried formulations (GG_5_ and GM_3_) were mixed with solid potassium bromide (KBr). The mixture was then pressed into a very thin pellet. The pellets were placed in the holder directly in the IR laser beam. Spectra were recorded using Shimadzu FTIR-8400s loaded with IR solution version 1.2 software. IR spectra of drug and polymer physical mixture were compared with IR spectra of pure drug for any major interaction.

#### DSC Study

The samples (2.5 mg) were taken in the solid state in the pan and were compressed with a high-pressure press. DSC of the samples were recorded using Modulated DSC systems (Q 1000 TA equipped with software Pyris 6.0). Thermal scanning was carried out at a rate of 10°C min^−^1 (from 0 to 300°C)) and nitrogen purge 35 ml/min.

## RESULTS AND DISCUSSION

### 

#### Preparation of Formulations

The compositions of the various batches of the GTN mucoadhesive system are shown in table 1. Initial experiments showed that increasing the concentration of gellan gum, in preparations containing only gellan, beyond 0.06% w/v caused gelation upon cooling to 40°C (during stirring). The ionic content of acetate buffer of pH of 5.0 which was used as vehicle in this case, could have contributed to the gelation of gellan when used beyond 0.06%, so that beyond this concentration gellan could not be used, since it form gel during the preparation process itself. Therefore less than this concentration was used in this study. In the combination systems with sodium alginate or sodium CMC, the concentration of gellan was kept constant at 0.03%. A similar result for ciprofloxacin, from gellan-based formulations with an increase in polymer concentration, is reported (20). Sodium alginate and sodium CMC were used as mucoadhesive agents combined with gellan at the concentration of 0.2, 0.4, 0.6% w/v and 0.1, 0.25, and 0.5% w/v, respectively provided the defined fluidity of the liquid formulation.

#### Evaluation of the formulations

The physico-chemical properties of the prepared gatifloxacin formulations are shown in table 1. The drug content, clarity and pH of the formulations were found to be satisfactory and the formulations were liquid at both room temperature and when refrigerated. The two main pre-requisites of mucoadhesive systems are viscosity and gelling capacity (speed and extent of gelation). Moreover, a mucoadhesive system should preserve its integrity without dissolving or eroding for a prolonged period of time to facilitate sustained release of the drug to the ocular tissues. All the formulations showed instantaneous gelation when contacted with the gelation fluids (STF). However, the nature of the gel which was formed depended to the polymer concentration. In the case of gatifloxacin formulation batch GG_1_ showed the weakest gelation, due to the presence of minimal amount of gellan (0.015%).

#### Rheological studies

**Figure 1:**
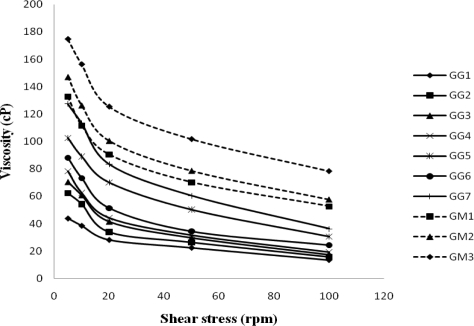
Rheological behavior of mucoadhesive system of gatifloxacin in non-physiological condition (pH, 5.0)

**Figure 2:**
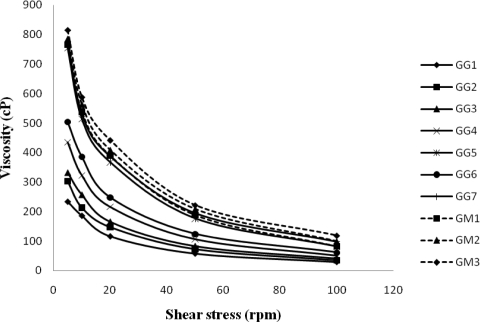
Rheological behavior of mucoadhesive system of gatifloxacin in physiological condition (pH, 7.2).

The administration of an ophthalmic formulation should not influence the pseudoplastic nature of the precorneal film, or the influence should be negligible (21). Figure 1 shows the viscosity of the developed formulation in a non-physiological condition (pH of 5.0). Figure 2 shows the viscosity of formulation in a physiological condition (pH of 7.2). All formulations either in non-physiological or physiological condition showed pseudoplastic behavior (viscosity that is high under a low shear rate and low under a high shear rate), which is fruitful for ophthalmic use due to the fact that the ocular shear rate is very high ranging from 0.03 s^−1^ during inter-blinking periods to 4250–28500 s^−1^ during blinking (22). If the viscosity at a high shear rate is too high, this will result in irritation. On the other hand, if the viscosity is too low, it will give rise to increased drainage. The pseudoplastic property of these formulations is in favour of sustaining drainage of drugs (from the conjunctival sac of the eye, without blinking difficulty in undergoing shear thinning). The viscosity of the formulations increased by increas in the concentration of the polymers.

#### Mucoadhesive evaluation

The viscosity of mucin colloidal dispersion is the net result of the resistance to flow exerted by individual chain segment, physical chain entanglement and the non covalent intermolecular interaction such as electrostatic, hydrogen and hydrophobic bonding (23, 24). These interactions are the identical forces involved in the process of mucin-polymer adhesion (25). Thus, force in a mucin bioadhesive system can be monitored by measuring viscosity. In fact, both physical and chemical bond energies in mucin-polymer interactions can be transformed into mechanical energy or work. This work causes changes in the shape or arrangement of macromolecules and is the basis of the viscosity changes (24).

The mucoadhesive forces of different formulations of gatifloxaicn mucoadhesive systems are shown in table 1. The mucoadhesive force (p<0.05) increased significantly as the concentration of sodium alginate and sodium CMC increased over the range of 0.2 to 0.6% and 0.1 to 0.5%, respectively. There was no significant difference between the mucoadhesive property of sodium alginate and sodium CMC (p>0.05). Formulation GG_5_ (Containing Gellan: Sodium CMC polymer ratio 0.03:0.5%) exhibited maximum mucoadhesive strength. The result also showed that the presence of sodium alginate or sodium CMC significantly increased the viscosity as well as the mucoadhesive property.

Gellan gum forms a clear gel in the presence of mono- or divalent cations. The electrolytes of the tear fluid especially Na^+^, Ca^2+^ and Mg^2+^ cations are particularly suited to initiate gelation of the polymer when instilled as a liquid solution into the cul-de- sac (26). Once gelled, the formulation resists the natural drainage process from the precorneal area. Residence at the site of drug absorption is prolonged and, subsequently, the bioavailability of the drug is increased (27).

Sodium carboxymethylcellulose (NaCMC), however, exhibits a mucoadhesive capacity comparable to that of poly(acrylic acid) (PAA) (28). It has an abundance of hydroxyl and ether groups along its length, which are responsible for mucoadhesive properties. Increasing the concentration of the sodium CMC in the formulation increased the bonds forming groups, thus increasing the mucoadhesicve force of the formulations (29). Mucoadhesion behavior of alginate was due to the low surface tension (31.5 mN/m) of the alginate, which was lower than the critical surface tension of the mucin coated cornea (38 mN/m), resulting in good spreading and adhesion (30).

#### In vitro release

The results clearly showed that the mucoadhesive systems have the ability to retain gatifloxacin in its matrix network and that the premature drug release will not occur.

#### The effect of gellan gum concentration on drug release

**Figure 3:**
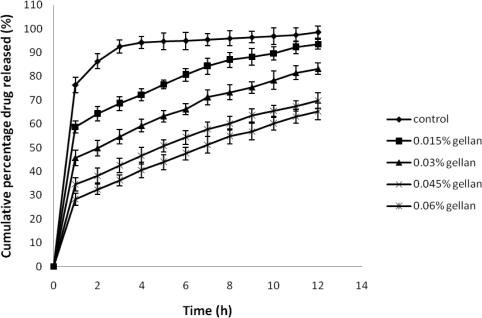
Effects of gellan gum concentration on the drug release Values are reported as mean±SD (n=3).

Gatifloxacin release from the control system was fast and the system was completely depleted the drug within 2 hrs. Gellan gum, an anionic polysaccharide, when incorporated as a part (0.015% to 0.06% w/v), controlled the release rate of the drug significantly (p<0.001) (Fig 3). Gellan gum undergoes gelation in the presence of cations, via a chemical bonding between the divalent cations and two COO^-^ groups of guluronic acid molecules in gellan chains (31). It is evident from the figure 3 that even at lower concentrations of gellan gum; the drug release was sustained for an extended period. Drug release seemed to slow down with an increase in gellan concentration. The release was significantly (p*<*0.001) slowed when the polymer concentration increased from 0.03% to 0.045% and 0.06%, while there was not much difference between the release patterns.

#### The effect of sodium carboxymethylcellulose concentration on drug release

**Figure 4:**
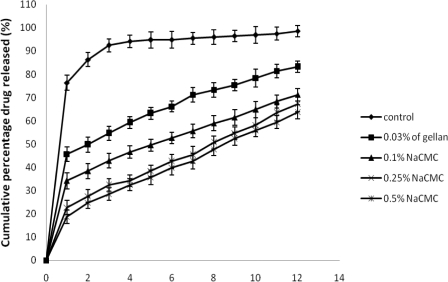
Effects of sodium CMC concentration on the drug release Values are reported as mean±SD (n=3)

Figure 4 shows the effect of sodium carboxymethyl cellulose on the drug release. Gellan at a concentration of 0.03% w/v was present in the formulation containing varying proportion of sodium carboxymethyl cellulose (GG_3_–GG_5_). The release of drug depends not only on the nature of the matrix, but also upon the polymer concentration. This may be due to structural reorganization of the hydrophilic polymer, sodium CMC. When sodium CMC is exposed to an aqueous medium, it undergoes rapid hydration and chain relaxation to form a viscose gelatinous layer (gel layer). Comparison of the release profile of GG_2_ (containing only gellan) with those of GG_3_ –GG_5_ indicate the burst effect was considerably reduced. Sodium carboxymethyl cellulose, was incorporated at 0.1% to 0.5% w/v affected the drug release significantly (p<0.001).

#### The effect of sodium alginate concentration on drug release

**Figure 5:**
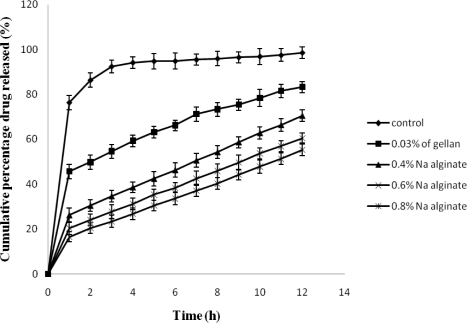
Effects of sodium alginate concentration on the drug release Values are reported as mean±SD (n=3)

Figure 5 shows the effect of sodium algiante on the drug release. Alginic acid is insoluble in water, but sodium alginate produces clear gels. Alginate is a natural block copolymer containing two types of monomers: beta-D mannuronic acid and alpha-L- guluronic acid. Alginates with a high guluronic acid content improve the gelling properties and reduce the total polymer to be introduced into the eye. The alginate forms 3-dimensional ionotropic hydrogel matrics, generally by the preferential interaction of calcium ions with the ‘G’ moieties resulting in the formation of a inhomogeneous gel (32). Gellan at a concentration of 0.03% w/v was present in the formulation containing varying proportion of sodium alginate (GM_1_–GM_3_). Results showed that the release retarding effect of sodium alginate was greater than sodium CMC. Sodium alginate, incorporated at 0.2% to 0.6% w/v significantly affected the drug release (p*<*0.001), thus indicating the additive effect of the formed calcium alginate on gel formation and consequently on drug release.

#### Kinetic Analysis

The correlation coefficient (*r*^2^) values for various release models viz., zero-order, first-order, and Higuchi models, were found. The *r*^2^ values suggest that the drug release from the mucoadhesive system predominately followed Higuchi's square root of time kinetics, as the values for *Q* vs. *t*^1/2^ (0.971–0.995) were always higher than those for *Q* vs. *t* (0.649–0.957) and log (100-Q) vs. *t* (0.85– 0.968). Release exponent, *n*, was *>*0.5 but *<*1, for all the batches indicating an anomalous or non-Fickian release and suggesting a coupled erosion- diffusion mechanism for the tested gatifloxacin mucoadhesive system.

#### Antimicrobial efficacy studies

#### Determination of minimum inhibitory concentration (MIC)

MIC determination was carried out using serial dilution method In GG_5_ formulation and pure drug sample the MIC concentration was found to be 0.15 and 0.075 µg/ml against *S.aureus* and *E.coli*, respectively. It showed that gatifloxacin retain its antimicrobial efficacy when formulated as a mucoadhesive system.

#### In vitro antibacterial activity

**Figure 6:**
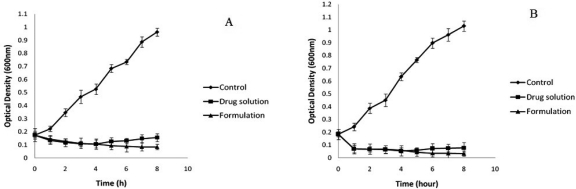
In vitro antibacterial activity of drug solution and mucoadhesive system (GG_5_). A - Activity against *E. coli*; B - Activity against *S. aureus* Values are reported as mean±SD (n=3)

Figure 6 shows the in vitro antibacterial activity of free GTN and GTN mucoadhesive system against *E. coli* and *S.aureus*. A significant decrease in OD600 value was observed in comparison with control (p*<*0.001). In the control set the OD600 value of *E.coli* and *S.aureus* increased with time.

In the control set the number of viable organisms increased with time. The control group was treated not any antimicrobial agent. Thus under favorable conditions (temperature and nutrient) the test organisms exhibited a significant growth. The growth of organisms was reduced or inhibited in the presence of either the solution form or the formulation as it was evident from the growth curve. Although no significant difference was observed between the antimicrobial activity of the drug and the formulation (p*>*0.001), a marginal increase of the viable count was observed in sample tubes containing drug solution after ∼4 hrs, while no increase in the viable count was observed during the 8 hrs incubation with formulation. These results showed the sustained release characteristics of gatifloxacin mucoadhesive system, that released GTN inhibits bacterial growth for a longer period.

#### IR Study

**Figure 7:**
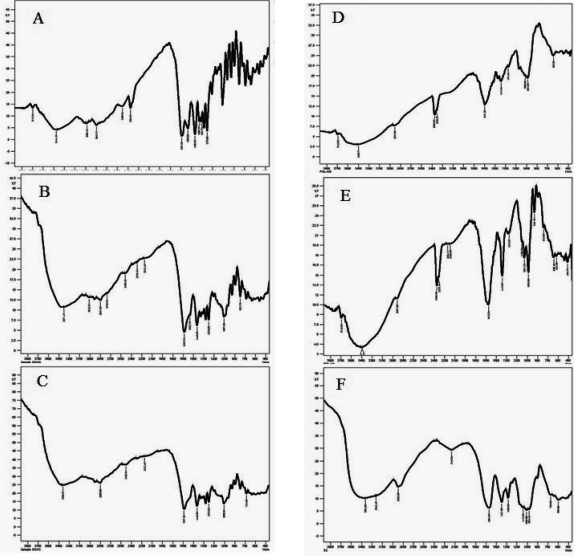
IR Spectrum of pure gatifloxacin, polymers and freeze dried formulation. A - Drug; B - freeze dried formulation (GG_5_); C - freeze dried formulation (GM_3_); D - Gellan gum; E - Sodium alginate; F-Sodium CMC

IR spectrums were recorded for pure gatifloxacin, polymers and freeze dried formulations (Fig 7). In both cases it was observed that the characteristic bands did not shift appreciably, suggesting the lack of any interaction between the drug and excipients.

#### DSC Study

**Figure 8:**
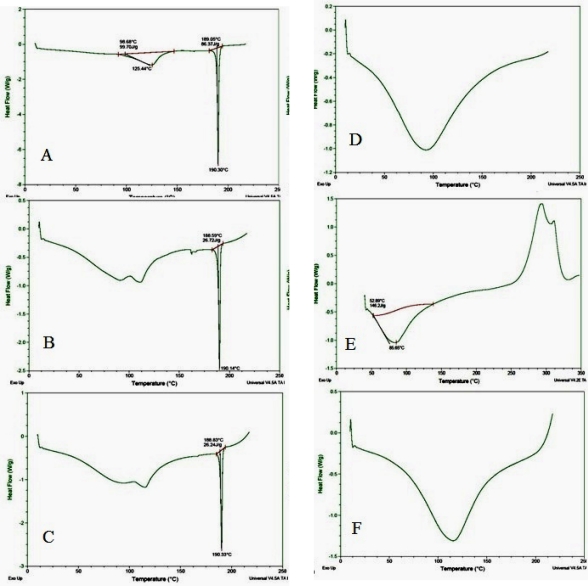
DSC endotherm of pure Gatifloxacin, polymers and freeze dried formulations. A - Drug; B - freeze dried formulation (GG_5_); C - freeze dried formulation (GM_3_); D - Gellan gum; E - Sodium CMC; F-Sodium alginate

DSC thermograms were recorded for pure gatifloxacin, polymers and freeze dried formulations (Fig 8). In both cases it was observed that the characteristic endotherm (corresponding to melt of the drugs) did not shift appreciably, suggesting the lack of any interaction between the drug and excipients.

## CONCLUSION

Gatifloxacin was successfully formulated as a mucoadhesive system using gellan combination with sodium carboxymethyl cellulose and sodium alginate. In vitro antibacterial activity of GG_5_ was evaluated against *S. aureus* and *E. coli,* in vitro. The formulated systems provided sustained release of the drug over a 12 hrs period in vitro. A Significant reduction in total bacterial count between drug solution (control) and mucoadhesive system was noted with both two organisms. However, GG_5_ effectively inhibited the growth of bacteria due to the sustained release characteristics of hydrogels. The developed formulation is a viable alternative to conventional eye drops for its ability to enhance bioavailability through its longer precorneal residence time and its ability to sustain the release of the drug. Also easy and decreased frequency of administration results in better patient compliance.
